# Fish optimize sensing and respiration during undulatory swimming

**DOI:** 10.1038/ncomms11044

**Published:** 2016-03-24

**Authors:** O. Akanyeti, P. J. M. Thornycroft, G. V. Lauder, Y. R. Yanagitsuru, A. N. Peterson, J. C. Liao

**Affiliations:** 1 Whitney Laboratory for Marine Bioscience, Department of Biology, University of Florida, Gainesville, Florida 3261, USA; 2 Department of Organismic and Evolutionary Biology, Harvard University, Cambridge, Massachusetts 02138, USA

## Abstract

Previous work in fishes considers undulation as a means of propulsion without addressing how it may affect other functions such as sensing and respiration. Here we show that undulation can optimize propulsion, flow sensing and respiration concurrently without any apparent tradeoffs when head movements are coupled correctly with the movements of the body. This finding challenges a long-held assumption that head movements are simply an unintended consequence of undulation, existing only because of the recoil of an oscillating tail. We use a combination of theoretical, biological and physical experiments to reveal the hydrodynamic mechanisms underlying this concerted optimization. Based on our results we develop a parsimonious control architecture that can be used by both undulatory animals and machines in dynamic environments.

## Abstract (web summary)

Head movements of swimming fishes are thought to exist only because of recoil of the oscillating tail. Here, Akanyeti *et al*. show that by controlling the timing of head movements, fish can improve their swimming efficiency while simultaneously optimizing sensing and respiration.

## Graphical abstract

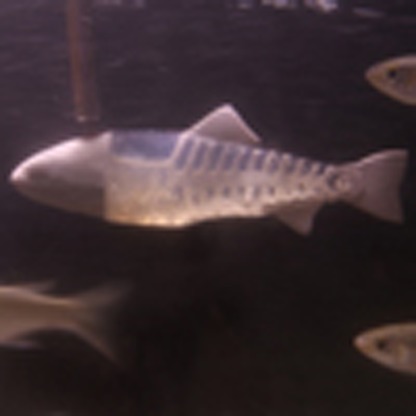

One of the most fascinating yet least understood attributes of living systems is their ability to simultaneously coordinate vital physiological functions. Organismal behaviour emerges from a multitude of tradeoffs that typically precludes the optimization of any one function. Undulation of the axial body is the fundamental motor pattern in vertebrates that predates the origin of paired fins and jaws, and powered the locomotion of the earliest animals with backbones. Compared with the body, movements of the head during undulation are far less understood. The long-held assumption is that head movements are the undesired by-product of swimming, where side forces produced by an oscillating tail lead to recoil at the anterior body^1^,^2^,^3^. Several morphological adaptations (for example, narrow necking at the tail, and lateral compression at the head) observed in tunas and bluegill sunfish have been suggested to reduce recoil^4^,^5^,^6^. Many studies have focused on the amplitude of head yaw without considering its timing with respect to body movements^1^,^3^,^7^,^8^,^9^,^10^, when in fact both must be taken into account to calculate resistive drag forces induced by the fluid^7^,^8^,^9^,^10^,^11^,^12^. In this study, we discover that by controlling the timing of head movements, fish can improve their swimming efficiency while simultaneously optimizing sensing and respiration.

## Results and Discussion

### Head movements increase swimming efficiency

**Figure 1:**
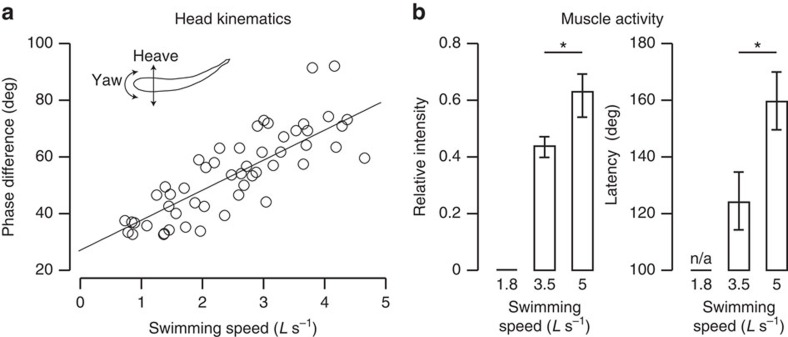
Kinematics and muscle activity for head motions of steadily swimming rainbow trout as a function of swimming speed. (**a**) The phase difference between yaw and heave increases linearly as a function of swimming speed (*y*=8.7*x*+37.6, *R*^2^=0.63, *P*<0.01, *n*=8 fish). We decomposed lateral head movements into angular rotation (yaw) and side to side motion (heave). The phase difference describes the timing between these two periodic motions. For instance, when the head is heaved to one extreme, 0° indicates that it is also yawed maximally on the same side. (**b**) Anterior red muscle activity (relative intensity=rectified area of a muscle burst) increases with swimming speed. Note that there is no anterior red muscle activity at 1.8 *L* s^−1^. Latency (timing of muscle activity relative to maximum head angle) increases with swimming speed. *P<0.05, unpaired *T*-test.

In freely swimming fish, we found that the timing (measured as phase difference) between yaw and side-to-side movements of the head increases with swimming speed (Fig. 1a). Simultaneous muscle recordings revealed that this kinematic pattern was correlated to anterior red muscle activity around the head at high swimming speeds (Fig. 1b).

We first set out to determine how head movements impact the propulsive efficiency of undulation. Measuring locomotor forces on a freely swimming fish is non-trivial^13^,^14^, and theoretical methods used to estimate performance do not reveal the individual contribution of head movements^15^. To circumvent this, we fabricated a flexible fish model where undulatory movements were generated from a single actuation point located just posterior to the head. This allowed us to evaluate performance in terms of thrust production, propulsive efficiency and swimming kinematics.

**Figure 2:**
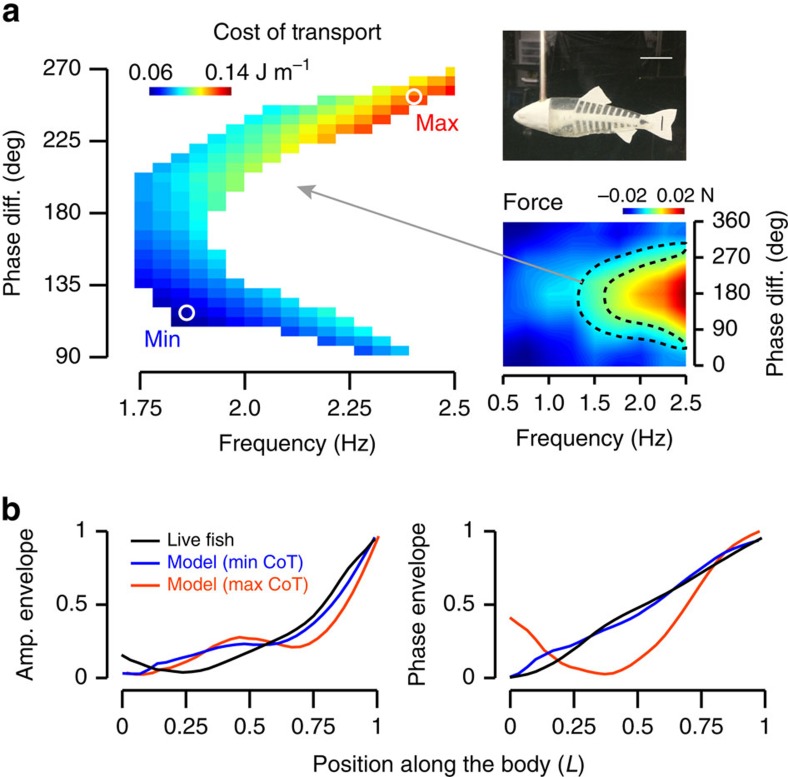
Efficient propulsion by an actuated, flexible fish model (top right image, the length of the scale bar is 3 cm) emerges when the yaw and heave of the head are coupled with the correct phase angle. (**a**) Force production of the model (lower right plot) was evaluated as a function of oscillation frequency and phase difference between yaw and heave at 0.8 *L* s^−1^. The heat map denotes the magnitude of force averaged over one tail-beat cycle. Negative values (blue) indicate a region where the fluid resistance was greater than the propulsive force generated by the model (drag). Positive values (red) indicate where the propulsive force of the model was greater than the fluid resistance (thrust). In a steadily swimming fish, there is no net force acting on the body (that is, thrust equals drag). In our experiments, this condition corresponded to the C-shaped region delineated by the dashed lines. The new heat-map plot on the left shows that within this region, the cost of transport differs as a function of oscillation frequency and phase difference. Low (blue) and high (red) cost of transport denote high- and low-propulsive efficiency, respectively. The locations of minimum and maximum values are shown (white circles). (**b**) The model displays very different kinematics depending on which phase difference and oscillation frequency values it adopts. At 110° (blue line, high-propulsive efficiency) it is similar to the amplitude and phase envelope of a live fish (black line). As in fish, body amplitude of the physical model increases posteriorly and the mechanical body wave is initiated at the head and travels down the body with a constant velocity. This is indicated by a linear increase in phase values down the body. At 270° (red line, low propulsive efficiency), the kinematics departs substantially from a live fish. Amplitude values are normalized to the maximum tail beat amplitude. Phase values are normalized to the phase difference between head and the tail.

Experiments with our physical model showed that coupling head and body movements with the correct phase angle generates efficient, fish-like propulsion. When undulatory body waves were created by heaving the model from side to side, we saw that additional head yaw can substantially alter the bending movements of the body, depending on the phase angle. For instance, no bending occurs at a phase angle of 0°, suggesting that the moments generated by the two motions interact destructively. Thrust-producing, undulatory waves occur between 60° and 300°. Among various options that would result in the same swimming speed, we discovered that choosing the appropriate phase angle (which fell within the range displayed by live fish) minimizes the power consumption by over 50% (Fig. 2a). At this phase angle, the swimming kinematics of the model are similar to those in live fish (Fig. 2b). The reduced power consumption may be explained by the following two, complementary mechanisms: (i) resonance as depicted in refs 16, 17 and (ii) constructive or destructive interactions of heave and yaw movements. Further analysis is required to better understand how these mechanisms interact with each other as a function of body mechanics and fluid environment.

### Enhanced lateral line sensing and respiration

Although head movements during undulation favour efficient propulsion, its consequences on flow sensing and respiration remain overlooked. Unlike the relatively simple pressure distribution around the straight body of a gliding fish^18^, the pressure distribution around an undulating fish that arises from complex fluid–structure interactions is not well understood. We first identified the relationship between head kinematics and undulation-generated pressures by developing a mathematical model using unsteady, potential flow theory. We validated the model by accurately predicting the pressures experienced by the physical fish model and freely swimming fish, irrespective of swimming speed (Supplementary Figs 1 and 2). Once the model was validated, we used it to examine how head movements of a live fish influenced lateral line sensing and respiration.

The lateral line system in fishes is an important sensory modality used during rheotaxis, prey detection and predator evasion^19^,^20^,^21^. This sensory system consists of mechanoreceptors distributed around the body, which provide information on local flows and pressure gradients^22^. It has long been presumed that the ability of lateral line to detect external stimuli is hindered by the self-generated stimuli of swimming, and that fish have two ways to deal with this problem: (i) by using an efferent system to filter out self-generated noise^23^, or (ii) by minimizing head and body motions, as when gliding or remaining stationary.

**Figure 3:**
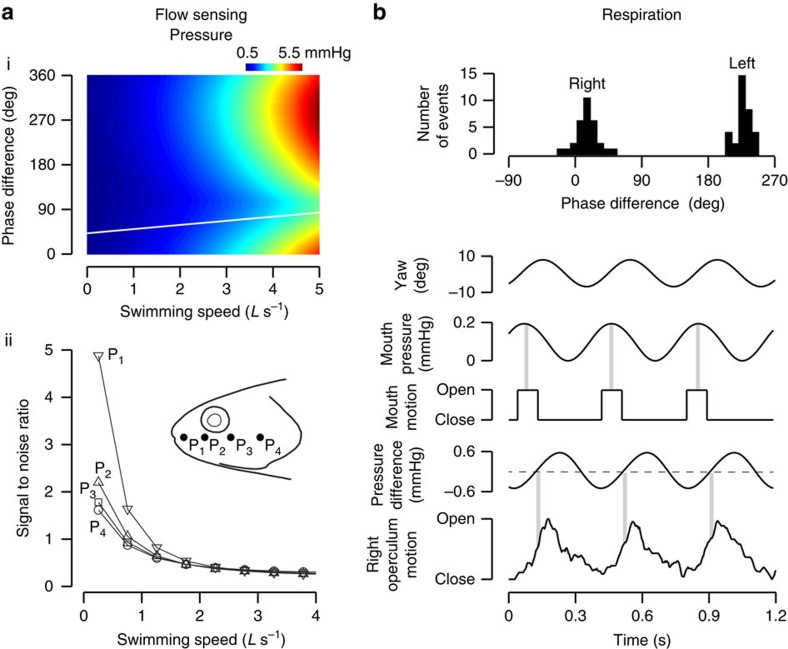
The importance of head movements on flow sensing and respiration, as revealed by a mathematical model that predicts pressure around the head during undulation. (**a**(i)) Swimming fishes minimize pressure around the head, which maximizes the ability for flow sensing. The intensity of self-generated pressures at the head (shown as a heat map) for all swimming speeds and phase differences between yaw and heave, where warmer colours indicate higher pressure. Self-generated pressures are comparable with the pressures generated by natural stimuli, which are typically between 0 and 2 mm Hg (refs 22, 49, 50). Pressure values were averaged across four points along the head. The phase difference values observed in live fish coincide with the region where pressure is at a minimum (the regression obtained from live fish in Fig. 1a is shown as a white line). (ii) The signal-to-noise ratio (pressure generated by an external stimulus divided by self-generated pressure) for different locations along the head. The intensity of self-generated pressures varies spatially around the head. Undulation-generated pressures at the head are lowest at the snout and increase posteriorly, in complete contrast to the pressure distribution found on a gliding fish. This pressure pattern in undulating fish makes them more able to detect external stimuli at the anterior part of the head. (**b**) Swimming fishes synchronize the movements of respiration and locomotion, which likely increases respiratory efficiency. A histogram of the phase difference between head yaw and opercula movements is shown in a live, freely swimming trout. The bimodal (as opposed to uniform) distribution of events confirms that respiration is tightly coupled to head movements. This pattern is consistent across three swimming speeds (1, 1.5 and 2 *L* s^−1^). The respiratory–locomotor coupling can occur either when the head is yawed to the right or left (Rayleigh test, *P*<0.01 in both cases). For example, when the head is yawed to the right, water first enters the mouth passively when the outside pressure is maximum, which occurs when the head is aligned with the swimming direction. In the second step, unidirectional flow past the gills is facilitated by opening the opercula when the pressure difference across the head is around 0 mm Hg, which occurs when the head is yawed maximally to the right. Vertical grey bars illustrate the alignment between undulation-generated pressures and respiratory movements.

We discovered that the motions associated with undulation can automatically enhance lateral line sensing on the head by minimizing self-generated stimuli. Fish move their heads in a way that minimizes pressure up to 50%, establishing a twofold greater sensitivity to an external stimulus than would otherwise be possible (Fig. 3a). At swimming speeds up to 2 *L* s^−1^, we found a heightened sensitivity around the anterior region of the head, which is where the majority of the encounters related to feeding and locomotion are initiated. We propose that during swimming, fish may not have to rely as extensively on the efferent system to distinguish between external and self-generated stimuli if they rotate their head in an appropriate phase with respect to side-to-side motion.

Swimming fishes must also maintain water flow across their gills to supply oxygen to their tissues. Fishes pump water through their gills by expanding and contracting the buccal cavity in concert with opening and closing the opercular valves. Initially, negative pressure produced by the expansion of the buccal cavity pulls water into the mouth, and positive pressure produced by the succeeding buccal contraction pushes the water out of the opercular valves^24^,^25^. In terrestrial animals with lungs, such as birds, horses and humans, respiratory–locomotor coupling is a well-established mechanism to enhance respiration during locomotion^26^. This coupling has not been demonstrated in fishes using undulatory propulsion, likely because respiration and undulation have historically been viewed as two independent processes. Given that the respiratory system is located in the head and the locomotory system is associated with the trunk, it is not unreasonable to assume that respiration and swimming would be decoupled. The contemporary view point is that the origin of the lung enabled respiratory–locomotor coupling to evolve in terrestrial animals^27^.

Here, we discover that fishes swimming with body undulations also show respiratory–locomotor coupling. Our pressure model reveals that undulation-generated pressures around the mouth and opercula oscillate dramatically. We found that fishes exploit these pressures by timing their respiratory movements accordingly, which likely minimizes the energetic cost of pumping the dense medium of water. High-speed, high-resolution video reveals that respiratory movements are tightly synchronized with head movements (Fig. 3b). When the pressure difference between the outside and inside of the mouth reaches 0.2 mm Hg, fishes open their mouth to allow water to flow in passively. Perhaps not coincidentally, this exact pressure difference is generated by the active buccal expansion of stationary fish^24^,^28^. In this way, we hypothesize that swimming fishes exploit self-generated pressures to circumvent the work of buccal pumping. Reduced expansion of the buccal cavity during steady swimming supports this hypothesis. Furthermore, the timing of opercular opening in relation to the outside pressure is critical, as it determines the symmetry of flow past the gills. Our data suggest that the opercula open when the pressure difference across the head is close to zero. This would ensure that flow occurs evenly across left and right side gills, which may be important for efficient oxygen uptake. Note that our analysis is based on the timing of the expansion of the opercular chamber, not the opening of the opercular slit. As an alternative hypothesis, the opercular slit may open when the pressure is least on one side (therefore maximal on the other side). The amount of work required for opercular opening is minimized for one side, but the binomial distribution in our data indicates that no particular side is favoured over the long term. A third hypothesis is that if the timing of mouth and opercula opening is synchronized, fish would only need to control the mouth as the opercula would follow passively. This respiratory–locomotor coupling in undulatory fishes was confirmed across several clades of ray finned fishes living in both fresh and salt water that occupy vastly different ecological niches.

### Pressure-based control of swimming

Although we demonstrate that head movements during axial body undulation simplify control by uniting propulsion, sensing and respiration, we were ultimately interested in how head movements are controlled during swimming. Fishes must continuously incorporate sensory feedback to adapt to a changing environment, such as when they change speed or recover from a hydrodynamic perturbation. Here, we propose a control architecture based on our experimental results, which shows that fishes can achieve this regulation by using solely their lateral line system.

**Figure 4:**
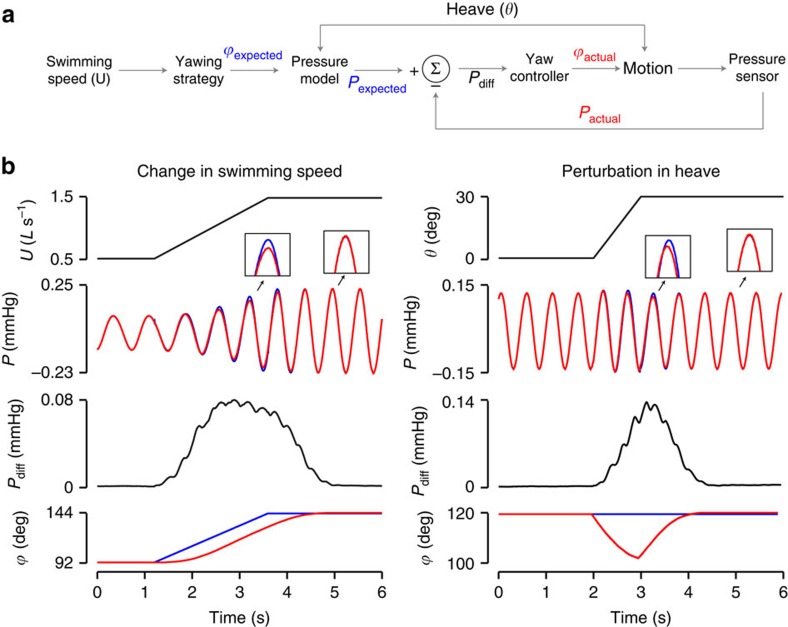
Proposed control architecture that universally regulates head movements of undulatory animals and machines in dynamic situations. (**a**) Model to control head movements in order to operate at optimal phase angles during steady swimming. (**b**) A demonstration of this control architecture is seen for two simulations of natural behaviours: (i) a change in swimming speed (left column) and (ii) a perturbation in heave motion (right column). When fishes change their swimming speed or are exposed to a lateral perturbation, a difference between the expected (from experience) and measured pressure (lateral line sensing) is established. A gradient descent algorithm uses the pressure difference to adjust the phase angle iteratively. As phase angle approaches the target value, the pressure difference, and hence phase adjustments, decreases. This process repeats until the phase angle matches the target value. In both cases, a discrepancy between the desired and actual pressure initiates a corrective response in head movements in order to reach a stable behaviour. *U*, swimming speed; *θ*, phase angle of heave; *ϕ*, phase difference between yaw and heave; *P*, pressure; P_diff_, pressure difference.

Our control architecture incorporates local pressure cues to generate desired head movements. For every swimming speed the target phase angle, which results in efficient propulsion, is associated with a distinct pressure profile. In live fish, this profile could be predicted by a neural representation based on experience that links head kinematics to pressure sensing, much like our pressure model. When a fish is not operating at the target phase angle, there is a difference between the expected and measured pressure, which is fed into a gradient descent algorithm to update the phase angle. As phase angle approaches the target value, the pressure difference gets smaller, as do the adjustments. This iterative process continues until the phase angle matches the target value (Fig. 4). The power of this simple control architecture is that it can be universally applied to any size and species of undulating fish, as well as to autonomous, underwater vehicles.

Life requires the successful, simultaneous execution of basic physiological functions. The coordination of these functions usually relies on distinct neural networks that run in parallel^29^,^30^. Over the past several decades, a number of studies have demonstrated that the passive mechanical properties of the body can simplify individual functions, releasing them from the need for precise neural control^31^,^32^,^33^,^34^,^35^. Here, we show that during aquatic axial undulation, head movements can allow seemingly disparate but fundamental functions to be coordinated simultaneously without tradeoffs.

## Methods

### Animal care

Experiments were conducted on rainbow trout (*Oncorhynchus mykiss*), an ecologically and commercially important species found worldwide. All research protocols were approved by the Institutional Animal Care and Use Committee at the University of Florida. Trout were held in a 4,731 circular fresh water tank maintained at 15±1 °C (DS-4-TXV Delta Star Chiller, Aqua Logic) on a 12 h/12 h light/dark cycle and fed commercial trout pallets daily. All values shown are mean±s.e.m.

### Head kinematics

Trout swam at speeds between 0.5 and 5 *L* s^−1^ in a re-circulating flow tank (*L*=18.5±0.8 cm, *n*=8 fish). We recorded swimming kinematics with two synchronized high-speed, high-resolution cameras (ventral and side view) at 250 frames per s. For each individual, five trials were conducted at each swimming speed, with each trial consisting of a three tail-beat sequence. We used customized scripts to extract the body midlines and analyse the data (Matlab, Mathworks). We quantified head movements by fitting a straight line to represent the region between the snout and the base of the cranium (that is, head line). We calculated the yaw as the angle between the head line and the axis of the swimming direction, and heave as the side to side motion of the head line. We used a sinusoidal motion to model both heave and yaw, and from this calculated the phase difference. To identify the relationship between phase difference and swimming speed, we ran a linear regression on data for all fish and report the *R*^2^ value.

### Electromyography (EMG)

EMG experiments were conducted by following the same protocol as previous work^36^. Briefly, two EMG electrodes were inserted into the superficial, axial red muscles on either side of the head. Experiments were conducted at three speeds (1.8, 3.5 and 5 *L* s^−1^), during which we recorded simultaneously EMG and kinematics data at sampling frequencies 4,000 Hz and 250 frames per s, respectively. For each individual, five trials were conducted at each speed. Each trial consisted of a four tail-beat sequence. We filtered the EMG data using a moving average (window size=0.025 s) and calculated the onset, relative intensity and duration of each muscle burst. We measured the relative strength of each burst as a product of its relative intensity and duration. The relative intensity was calculated as the mean spike amplitude for the rectified muscle burst and normalized by the maximum mean spike amplitude.

For every tail beat, we computed the time delay between the onset of muscle activity and maximum head angle (latency) from the kinematics data. We used an unpaired *T*-test to evaluate whether the measured variables were significantly different between medium and high speeds (there was no muscle activity at the lowest speed). Data were collected from five trout, although our data analysis focused on comprehensive data sets from two individuals (*L*=16.2 and 16.8 cm).

### Respiratory–locomotor coupling

Buccal pumping data were taken from video sequences when fish swam less than 2 *L* s^−1^ (70 events analysed from five individuals). At higher swimming speeds, fish transitioned to ram ventilation, as seen previously^37^. We manually digitized head, mouth and opercular movements. We used a cross-correlation method to calculate the phase difference between head and opercula movements, from which we evaluated the synchronicity between respiration and locomotion using a Rayleigh test. We tested our hypothesis on respiratory–locomotor coupling with several other actinopterygian species, including jack crevalle (*Caranx hippos*), blue fish (*Pomatomus saltatrix*), red drum (*Sciaenops ocellatus*), striped mullet (*Mugil cephalus*), black drum (*Pogonias chromis*) and spotted sea trout (*Cynoscion nebulosus*).

### Physical fish model

We obtained a preserved rainbow trout specimen from the Florida Museum of Natural History (lot #99345) and scanned the body using a ZScanner 700 (Z Corporation^R^). We reconstructed a three-dimensional (3D) Computer aided design (CAD) model from the scanned images using Rhinoceros (v5) and Meshlab (v1.3.3) software. From this, we made a bio-inspired physical model, which consisted of a rigid head, flexible (but not articulated) backbone and a soft body. The backbone included a vertebral column with inclined neural and haemal spines (∼30°) and median fins (caudal, anal and dorsal). The head, backbone and fish mold were 3D printed with Acrylonitrile butadiene styrene (ABS) plastic using a Makerbot Replicator 2X (MakerBot^R^ Industries LLC). We placed the printed head and backbone into the mold and injected liquid plastisol to fill out the rest of the body (LureCraft Inc.). This modular, multi-material design allowed us to iteratively adjust the stiffness of the model by changing the backbone thickness and body compliance until we arrived at fish-like motions. The final model had a total length of 18 cm. The width of the backbone was 0.1 cm and the height was 0.7 cm except near the head, where it was 2 cm to account for the large bending forces generated by the head.

### Propulsion experiments

We measured the performance of the physical fish model in a re-circulating flow tank at Harvard University. The model was connected to a robotic controller equipped with an ATI Nano-17 six-axis force/torque transducer (ATI Inc.) via an 8-mm stainless steel rod^38^. The controller had two degrees of freedom, allowing us to simultaneously heave and yaw the model^39^. We used a sinusoidal motion for both degrees of freedom,


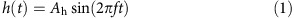



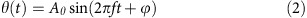


where we kept the amplitude of heave and yaw constant at *A*_h_=1 cm and *A*_θ_=10°, respectively. We evaluated the performance of the model at one flow velocity (0.8 *L* s^−1^) as a function of frequency and phase difference between heave and yaw (*f*=0.5–2.5 Hz in 0.25 Hz increments, *ϕ*=0°–360° in 30° increments). For all experiments, we recorded the propulsive forces and torques on the mounting rod over ten tail-beat cycles. From these measurements, we calculated thrust and power^40^. To evaluate the performance of the model during steady swimming, we identified combinations of actuation parameters where the thrust produced was equal to the drag imposed by the flow over a tail-beat cycle. To account for the drag of the rod, we repeated the experiments with the rod without the physical model and subtracted these values from the original experiments. We calculated the cost of transport (CoT) of each movement combination by dividing power by speed, where a low CoT denotes high propulsive efficiency. To estimate the error in our results, several experiments were chosen at random and repeated multiple times. The standard error in all cases was <5%.

### Comparison between swimming kinematics of live fish and the physical model

We identified the movement combinations that produced the lowest and highest CoT and recorded the swimming kinematics of the physical model with high-speed video (250 frames per s). After extracting the midlines for the whole body, we calculated the amplitude and phase envelope using a Fourier analysis^41^. We represented the lateral motion of each point along the midline with a periodic sine function. We estimated the parameters of the sine function (amplitude, frequency and phase) using a least square algorithm by minimizing the error between the actual and predicted motions in time. Given that all points along the midline oscillated with the same frequency, it was possible to analyse how amplitude and phase values changed along the body. We compared the amplitude and phase envelopes of the physical model to those calculated for live fish.

### Pressure model

We present a theoretical model to estimate the pressure distribution around a dynamically moving, rigid head (note that this model cannot be used to predict the pressure distribution for a flexible body). Under the assumptions of irrotational flow and zero boundary layer effects, the hydrodynamic pressure distribution around the head was approximated with an unsteady, potential flow equation,


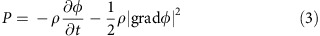


where *ρ* is the water density and *φ* is the velocity potential^11^. Velocity potential was written as,


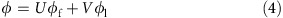


where *U* and *V* are forward and lateral velocity of the head, respectively. *φ*_f_ and *φ*_l_ are two scalar functions varied depending on the position of the head along forward and lateral directions, respectively. Expansion of the potential flow equation led to the candidate pressure model with nine terms,


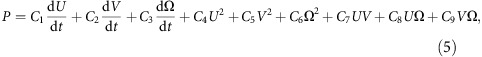


where Ω is the angular velocity of the head. The coefficients, *C*_1_–*C*_9_, indicate the contribution of each term and depend on the geometry of the head. We followed a data-driven approach to estimate the coefficients based on experimental measurements, as it was difficult to derive the coefficients theoretically.

### Pressure experiments with the physical fish model

Our 3D printed heads were designed with four holes arranged from snout to operculum along a horizontal line. In each hole, we embedded a surgical grade, 1 mm tip diameter micro-pressure transducer (Millar Co.). We actuated the physical model as previously, but across a greater range of speeds (from 0 to 2 *L* s^−1^, in 0.25 *L* s^−1^ increments). We simultaneously recorded pressure and kinematics data using Labview (National Instruments) at 1,000 Hz. We calculated the variables *U*, *V* and *Ω* from the kinematics data. We filtered the pressure data using low- and high-pass filters with 1 and 20 Hz cutoff frequencies, respectively. Overall, our data included a time series of three kinematics variables (input) and four pressure measurements (output). We split the data into two halves, consisting of (i) a training data to estimate the coefficients of the model and (ii) a validation data to evaluate the performance of the model.

### Training and validation of the pressure model

We estimated the coefficients of the pressure model using an orthogonal parameter estimation algorithm^42^. One advantage of orthogonal parameter estimation algorithm over classical least square methods is that it provides an indication to the significance of the model terms. This allows the removal of insignificant terms and yields more parsimonious models^43^,^44^. First, an auxiliary model was defined such that the terms in the model were orthogonal over the training data set. The coefficient of each term in the auxiliary model was then estimated using the least squares method. The individual contribution of each term to the desired output variance was measured using an error reduction ratio^42^. The terms with contributions less than a predetermined threshold were removed from the model, and the coefficients of the remaining terms were re-computed. This iterative process continued until the auxiliary model passed the model validity test^45^. We transferred the auxiliary model from orthogonal to Euclidean space to derive the actual pressure model. We arrived at the final pressure model,


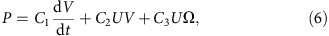


where *C*_1_–*C*_3_ vary depending on the position along the head (Supplementary Table 1). We evaluated the performance of the model using the validation data set by comparing the measured and predicted pressure. In particular, we computed the mean absolute difference and correlation coefficient.

### Pressure measurements on freely swimming fish

In addition, we tested the predictive power of the pressure model on freely swimming fish (*n*=5 fish). To do this, we developed a technique to outfit a freely swimming fish with pressure transducers (the same sensors as used in the physical model experiments). Three transducers were attached along the skin of the fish using suture thread (one at the snout, one on the left opercula and one on the right opercula). After recovery from surgery, trout swam in the flow tank at speeds between 1 and 5 *L* s^−1^. We used a Powerlab 16SP analogue-to-digital converter (ADInstruments) to record pressure signals at a sampling frequency of 1,000 Hz. We simultaneously measured the swimming kinematics using a high-speed camera at a sampling frequency of 250 frames per s. The pressure data were pre-processed as previously described. We computed the kinematic variables *U*, *V* and Ω and entered them into the pressure model. We then compared the measured pressure to the predicted pressure. To evaluate whether the pressure transducers interfered with the natural swimming movements of the fish, we compared the swimming kinematics of the fish with control data and did not find any significant differences.

### Self-motion effects on lateral line sensing

Recording from the lateral line of freely swimming fish is exceedingly difficult with current technology. Because self-generated pressures have a direct relationship to neuromast deflection and hair cell activity^46^,^47^, we used the pressure model to evaluate how pressure distribution around the head is influenced by the phase difference between heave and yaw movements of the head. For swimming speeds between 1 and 5 *L* s^−1^, we simulated the pressure distribution around the head as a function of phase difference (0°–360° in 5° increments). For each speed, we kept the kinematic variables (oscillation frequency, heave and yaw amplitudes) identical to those observed in freely swimming fish.

### Pressure generated by external stimuli

To better evaluate if the magnitude of self-generated pressure values would be meaningful to live fish, we compared self-generated pressure values to those generated by an external stimulus found in the environment. We approximated these pressures as a dipole source,


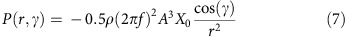


where *r* and *γ* are, respectively, the relative distance and angle from the stimulus, *f* and *X*_0_ are, respectively, the oscillation frequency and amplitude of the stimulus, and *A* is the size of the stimulus^48^. The values chosen for a biologically realistic stimulus were *r*=0.6 *L*, *γ*=0°, *f*=6.7 Hz, *a*=0.2 *L*, *X*_0_=0.02 *L*. We calculated the signal-to-noise ratio as the ratio between the pressures generated by external stimuli (signal) and self-generated pressures (noise).

### Control architecture and simulations

For a given swimming speed and heave motion, phase difference between yaw and heave of the head, *ϕ*, is controlled using a gradient descent algorithm


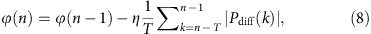


where *n* represents discrete time steps, *P*_diff_ is the pressure difference between measured and desired pressure, *η* is the learning rate and *T* is the time interval to calculate the mean pressure difference. Phase difference is updated iteratively at every time step where learning rate and time interval determine the reaction rate to the detected pressure difference. Two numerical simulations were carried out in Matlab: simulation 1 (change in swimming speed): *η*=1, *T*=one tail-beat cycle, and simulation 2 (perturbation in heave): *η*=0.8, *T*=one tail-beat cycle. Values for *η* and *T* were derived empirically.

## Additional information

**How to cite this article:** Akanyeti, O. *et al*. Fish optimize sensing and respiration during undulatory swimming. *Nat. Commun.* 7:11044 doi: 10.1038/ncomms11044 (2016).

## Supplementary Material

Supplementary Information Supplementary Figures 1-2 and Supplementary Table 1.
