# Supplementary material for: Fish optimize sensing and respiration during undulatory swimming
Source: Nat Commun. 2016 Mar 24;7:11044. doi: 10.1038/ncomms11044 (PMC4820825; doi:10.1038/ncomms11044)
Supplement: Supplementary Information — Supplementary Figures 1-2 and Supplementary Table 1. [file ncomms11044-s1.pdf]

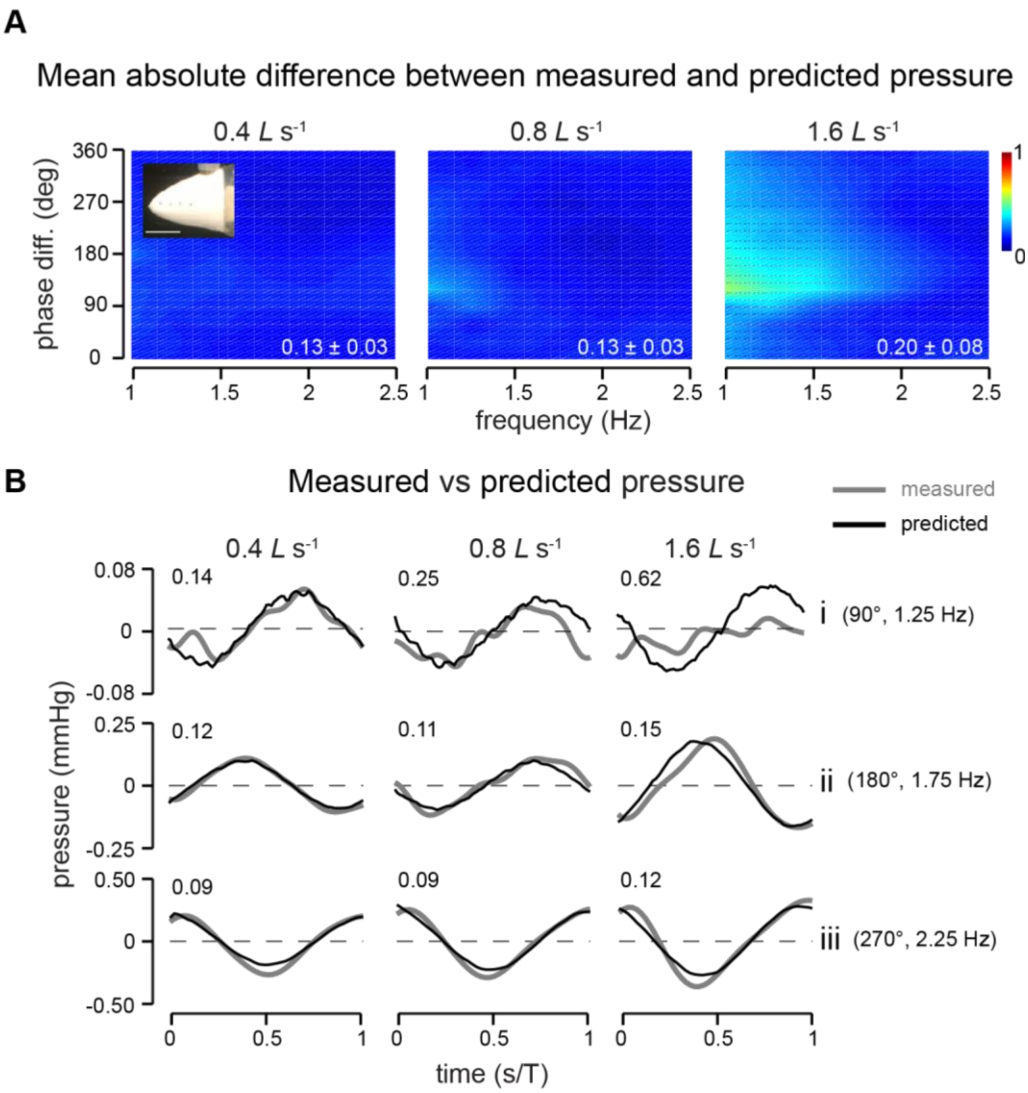

Supplementary Fig 1. Experimental validation of the theoretical pressure model using the physical fish model (inset image). **A**. Heat map plots illustrate the absolute difference between measured and predicted pressure, averaged over one tail beat cycle, at three flow speeds (0.4 L s^{-1}, 0.8 L s^{-1} and 1.6 L s^{-1}). Values are normalized to the maximum amplitude of the measured pressure to emphasize relative differences. The overall performance value for the model is shown at the bottom right for each plot. The length of the scale bar is 1cm. **B**. To illustrate the temporal performance of the model, we show measured (black) and predicted (gray) pressure over one tail beat cycle (T) for three phase difference and oscillation frequency values (**i**) (90°, 1.25 Hz), (**ii**) (180°, 1.75 Hz) and (**iii**) (270, 2.25 Hz). Numbers on the top left corner show the mean absolute pressure difference.

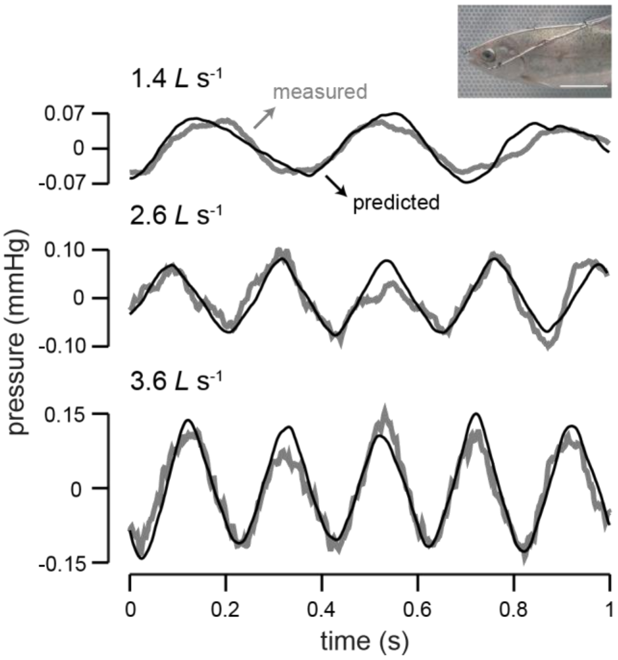

Supplementary Fig 2. Validation of the pressure model using live fish. The measured (black line) and predicted (gray line) pressure on the head of a freely-swimming fish outfitted with pressure sensors (inset image) at three swimming speeds. The mean absolute difference is less than 15% for all speeds. The length of the scale bar is 2 cm.

pressure model	C_1	C_2	C_3	mean absolute difference	correlation coefficient	p-val
P_1	0.03	3.90	-0.04	0.11	0.69	<0.001
P_2	0.07	4.23	-0.10	0.12	0.92	<0.001
P_3	0.09	3.95	-0.15	0.15	0.94	<0.001
P_4	0.10	3.09	-0.17	0.18	0.96	<0.001

Supplementary Table 1. Coefficients (C_1-C_3) and performance of the pressure model at four different locations (P_1-P_4) along the head (see schematic diagram of fish head in Fig. 3Aii).
